# Breast Cancer, Sickness Absence, Income and Marital Status. A Study on Life Situation 1 Year Prior Diagnosis Compared to 3 and 5 Years after Diagnosis

**DOI:** 10.1371/journal.pone.0018040

**Published:** 2011-03-30

**Authors:** Sonja Eaker, Annette Wigertz, Paul C. Lambert, Leif Bergkvist, Johan Ahlgren, Mats Lambe, on behalf of the Uppsala/Örebro Breast Cancer Group

**Affiliations:** 1 Department of Surgical Sciences, Uppsala University, Uppsala, Sweden; 2 Regional Oncologic Centre, Uppsala University Hospital, Uppsala, Sweden; 3 Centre for Biostatistics & Genetic Epidemiology, Department of Health Sciences, University of Leicester, Leicester, United Kingdom; 4 Department of Medical Epidemiology and Biostatistics, Karolinska Institutet, Stockholm, Sweden; 5 Department of Surgery and Centre for Clinical Research, Uppsala University, Central Hospital, Västerås, Sweden; 6 Department of Oncology, Gävle Hospital, Gävle, Sweden; 7 Centre of Clinical Research, Uppsala University/County of Gävleborg, Gävle, Sweden; Erasmus University Rotterdam, Netherlands

## Abstract

**Background:**

Improved cancer survival poses important questions about future life conditions of the survivor. We examined the possible influence of a breast cancer diagnosis on subsequent working and marital status, sickness absence and income.

**Materials:**

We conducted a matched cohort study including 4,761 women 40–59 years of age and registered with primary breast cancer in a Swedish population-based clinical register during 1993–2003, and 2,3805 women without breast cancer. Information on socioeconomic standing was obtained from a social database 1 year prior and 3 and 5 years following the diagnosis. In Conditional Poisson Regression models, risk ratios (RRs) and 95% confidence intervals (CIs) were estimated to assess the impact of a breast cancer diagnosis.

**Findings:**

Three years after diagnosis, women who had had breast cancer more often had received sickness benefits (RR = 1.49, 95% CI 1.40–1.58) or disability pension (RR = 1.47, 95% CI 1.37–1.58) than had women without breast cancer. We found no effect on income (RR = 0.99), welfare payments (RR = 0.98), or marital status (RR = 1.02). A higher use of sickness benefits and disability pension was evident in all stages of the disease, although the difference in use of sickness benefits decreased after 5 years, whereas the difference in disability pension increased. For woman with early stage breast cancer, the sickness absence was higher following diagnosis among those with low education, who had undergone mastectomy, and had received chemo- or hormonal therapy. Neither tumour size nor presence of lymph nodes metastasis was associated with sickness absence after adjustment for treatment.

**Interpretation:**

Even in early stage breast cancer, a diagnosis negatively influences working capacity both 3 and 5 years after diagnosis, and it seems that the type of treatment received had the largest impact. A greater focus needs to be put on rehabilitation of breast cancer patients, work-place adaptations and research on long-term sequelae of treatment.

## Introduction

Cancer survival has improved during the last decades, particularly for patients diagnosed with breast cancer[1], [2], the most common cancer in women. New and better combinations of treatments (chemotherapy, hormonal therapy, and radiotherapy)[3], [4], [5] as well as earlier detection through mammography screening programs[6], [7], [8] have contributed to the improved prognosis. However, cancer and its treatment may cause both physical and psychological problems that may influence ones future life situation. Several recent studies have also addressed issues related to employment and predictors of returning to work, such as the effect of age, education, type of job, workplace adaptations, adjuvant treatment and recurrences [9], [10], [11], [12], [13], [14], [15], or time to return to work [16], [17],[18], among women diagnosed with breast cancer. Other aspects of life in terms of marital status and risk of divorce after a cancer diagnosis have also been studied [19], [20], [21], [22] where both negative and no effect have been found. For deeper analysis we used the opportunity in Sweden to link information from several population based registers enabling us not only to investigate the effect of breast cancer on the future life situation in the long term (both 3 and 5 years after the diagnosis), but also to relate the findings to stage at diagnosis and the treatment received.

In the present study we used information retrieved through record linkage between three population-based registries to investigate possible socioeconomic consequences of breast cancer survivors in terms of sickness benefits, marital status, welfare dependence, and income changes 3 and 5 years following a breast cancer diagnosis. The investigation was made possible by comparing breast cancer patients with women without breast cancer randomly selected from the same population. We also investigated the possible influence of tumour characteristics and type of cancer treatment on the likelihood of returning to work.

## Materials and Methods

This study was based on a dataset generated by record linkage between three population-based registers, the Regional Quality Register of Breast Cancer of the Uppsala/Örebro Region in Central Sweden, the National Population Register, and the LISA-database (an integrated database for labour market research). Linkage was made possible by the individually unique National registration number assigned to each resident in Sweden at birth or time of permanent residency.

### Regional Quality Register of Breast cancer

The Regional Quality Register of Breast Cancer in the Uppsala/Örebro health care region covers seven counties in central Sweden with a source population of 1.9 million (representing about 20% of Sweden's total population). The main purpose of the register is to monitor the quality of care based on regional or national guidelines for breast cancer management. The register includes individual information reported continuously from the clinicians on date of diagnosis, detection mode, tumour-stage, tumour characteristics and primary surgical and oncological treatment for all newly diagnosed breast cancer patients. The database is continuously updated against the National Population Register to assess current vital status of the registered patients. The Regional Quality Register of Breast Cancer was started in 1992 and has been estimated to have a coverage of 97%, following a validation against the records of the mandatory Swedish National Cancer Register[23].

### The National Population Register

This register, which is the basic register of the population of Sweden, contains information about who lives in Sweden and place of residence. The register is managed by the Swedish Tax Agency[24].

### The LISA-database (Social Database)

Individual information on socioeconomic and demographic factors was obtained from the LISA-database managed by Statistics Sweden[25]. This nationwide database, which integrates existing data from registers in the labour market-, educational- and social sector, consists of data from 1990 and onwards on all individuals 16 years or older registered as living in Sweden. The database is updated on a yearly basis regarding individual information on educational level, income, socioeconomic index, welfare benefits and employment status.

### Study subjects and Follow-up

#### Study period

We investigated patients reported to the Uppsala/Örebro Regional Quality Register of Breast Cancer between January 1, 1993 and December 31, 2003.

#### Follow-up

Information from LISA was collected for the years 1992 (1 calendar year prior to the patients with the earliest diagnosis year) to 2006. This means that we had a follow-up of 5 years for all women diagnosed between 1993 and 2001, but a follow-up limited to 3 years for women diagnosed between 2002 and 2003 (Table 1).

**Table 1:** Patients 20–59 years of age with a breast cancer diagnosis between 1993 and 2003 matched with women without breast cancer by birth year, gender and community.

	1 calendar year prior to the diagnosis			3 calendar years after the diagnosis			5 calendar years after the diagnosis		
	Breast cancer	Not breast cancer		Breast cancer	Not breast cancer		Breast cancer	Not breast cancer	%
	No.	No.	%	No.	No.	%	No.	No.	%
Diagnosis year									
1993	321	1605	6.7	321	1605	6.7	321	1605	8.5
1994	373	1865	7.8	373	1865	7.8	373	1865	9.9
1995	411	2055	8.6	411	2055	8.6	411	2055	10.9
1996	407	2035	8.6	407	2035	8.6	407	2035	10.8
1997	411	2055	8.6	411	2055	8.6	411	2055	10.9
1998	489	2445	10.3	489	2445	10.3	489	2445	13.0
1999	455	2275	9.6	455	2275	9.6	455	2275	12.1
2000	433	2165	9.1	433	2165	9.1	433	2165	11.5
2001	475	2375	10.0	475	2375	10.0	475	2375	12.6
2002	446	2230	9.4	446	2230	9.4	-	-	-
2003	540	2700	11.3	540	2700	11.3	-	-	
Total	4761	23805		4761	23805		3775	18875	

#### Women with breast cancer

This group was defined as all women aged 20–59 years with a diagnosis of primary invasive breast cancer between January 1, 1993 and December 31, 2003 and reported to the Uppsala/Örebro Regional Quality Register of Breast Cancer. Since we were interested to investigate the consequences of cancer on those who we were able to follow for 5 years or more (or 3 years for women diagnosed between 2002 and 2003), women with incomplete follow-up were excluded. In the initial database of 5482 patients, 4413 were diagnosed between 1993 and 2001, and 1069 women between 2002 and 2003. Of the 4413 women diagnosed between 1993 and 2001, 3776 (85.6%) could be followed for 5 years, and of the 1069 women diagnosed between 2002 and 2003, 986 (92.2%) could be followed for 3 years. This means that from the initially identified 5482 patients, 721 were lost to follow-up and therefore excluded. The main reason for failure to follow-up was death, i.e. 94.8% (676 of 721) of the women had died within the follow-up period. The other main reason for lost follow-up was that the women had moved out of the health care region. Proportionally more women with advanced stages (III-IV) were lost to follow-up than were women in earlier stages, i.e. of the original sample 89% (4095/4587) of women with stages I-IIB could be followed compared to 64% (225/354) of the women with stages III-IV. Also, the women lost to follow-up were proportionally younger, i.e. of women younger than 44 years of age 83% could be followed (898/1080 = 83%) compared to 88% (3863/4394) of women aged 45-59 years (data not shown). In one prior study we also found that younger women had a poorer 5 year relative survival after breast cancer[26].

#### Women without breast cancer

Each patient was individually matched to 5 women without breast cancer (total 23805 women) registered in the National Population Register with complete follow-up by birth year, and community one year prior to the breast cancer patients' calendar year of diagnosis. The main reason for matching was to minimise confounding that may influence the outcome of investigated variables, such as working status and income. The matching was conducted by Statistics Sweden, and made possible by use of the National registration number (the individuals unique National registration number is registered in all registers used in this study and all information about an individual is attached to his/her unique number) and information available in the National Population Register (where also historical information on residential community is available).

### Information collected from the Registers

#### Regional Quality Register of Breast Cancer

We retrieved information on patients with early stage breast cancer classified according to tumour stage [UICC stages I, IIa, IIb], tumour-size in millimetre [1–10, 11–20, 21–50, 50+], lymph node involvement [positive, negative], primary surgical treatment [mastectomy, breast-conserving surgery (BCS), none or missing], and intended oncological treatment [radiotherapy, chemotherapy, hormonal therapy (Tamoxifen)]. We also investigated the effect of sentinel node biopsy and axillary dissection [sentinel node only, axillary dissection with 1–9 nodes examined, axillary dissection with more than 10 nodes, missing]. Since the sentinel node procedure not was fully introduced in our health care region until 2005, only few node-negative patients were staged with this method in the study cohort leading to low power and making it difficult to generalize results.

#### The LISA-database

From this register we retrieved individual information on all women 1 calendar year prior to the breast cancer patients diagnosis as well as 3 and 5 years after the year of diagnosis on education [low (compulsory school which is mandatory, ≤9 years,), middle (gymnasium/upper secondary school, 10–12 years), high (college and university, 13 or more years), unknown], working at least part-time [yes, no], unemployment [yes, no], retirement [yes, no], use of welfare [social welfare allowance and/or housing allowance, yes, no], marital status [married/registered partner, single (includes cohabitants without registered partnership), divorced, widowed] and number of people in the household [1, 2 or more].

#### Sickness absence

The social insurance system in Sweden covers everyone that resides or works in Sweden, and provides financial protection for persons with a disability or in connection with an illness. *Sick pay* can be obtained (from the employer) the first 14 days of a sick period. *Sickness benefit* is paid by the Swedish Social Insurance Agency if the employee is ill for a longer period than 14 days. The sickness benefit is approximately 80% of the individuals income up to a certain limit (a year wage of SEK 321 000). *Sickness pension* (or disability compensation) can be received if the work capacity is permanently reduced by at least a quarter. Full income-related sickness compensation provides 64% of the individuals assumed income. Sickness absence in our study was defined as all women registered during the year of follow-up with sickness benefit or disability pension at least part time [yes, no]. We also presented the values of sickness benefit [yes, no], and disability pension [yes, no].

#### Disposable income

The disposable income is the individual's total income (from all sources) minus taxation. To show possible differences in income 1 year prior to diagnosis between women with and without breast cancer we categorized income as lowest 25%, 25–50%, 50–75% and 75–100% (Table 2). To investigate changes in income after the disease, we compared each individual's income 3 and 5 years after the diagnosis with that 1 year prior to the year of diagnosis, i.e. we investigated whether they had had a 10% income increase or a 20% income increase.

**Table 2:** Education, working life, income and marital status 1 year before diagnosis among women diagnosed with breast cancer between 1993 and 2003 and women without breast cancer.

	1 year prior to diagnosis					
	Women with breast cancer		Women without breast cancer		Crude model	
	No.	%	No.	%	OR	95% CI
*Education*						
Low	1155	24.3	6434	27.0	1.00	(ref.)
Middle	2191	46.0	10828	45.5	1.13	1.05–1.23
High	1398	29.4	6451	27.1	1.21	1.11–1.32
Unknown	17	0.4	92	0.4	1.04	0.62–1.75
*Working (part-time or more)* 1						
Yes	4196	88.1	20637	86.7	1.14	1.04–1.26
No	565	11.9	3168	13.3	1.00	(ref.)
*Sickness benefit*						
Yes	944	19.8	4579	19.3	1.04	0.96–1.12
No	3817	80.2	19226	80.8	1.00	(ref.)
*Disability pension*						
Yes	572	12.0	3062	12.9	0.92	0.84–1.02
No	4189	88.0	20743	87.1	1.00	(ref.)
*Sickness absence* 2						
Yes	3395	71.3	16830	70.7	0.97	0.91–1.04
No	1365	28.7	6975	29.3	1.00	(ref.)
*Unemployment* 3						
Yes	694	14.6	3321	14.0	1.05	0.94–1.15
No	4067	85.4	20484	86.1	1.00	(ref.)
*Welfare* 4						
Yes	464	9.8	2609	11.0	0.87	0.78–0.97
No	4297	90.3	21196	89.0	1.00	(ref.)
*Disposable income*						
01–25%	1176	24.7	5982	25.1	1.00	(ref.)
26–50%	1127	23.7	6022	25.3	0.95	0.87–1.04
51–75%	1232	25.9	5898	24.8	1.06	0.97–1.06
76–100%	1226	25.8	5903	24.8	1.06	0.97–1.16
*Marital status*						
Married/Partner	3000	63.0	14968	62.9	1.00	(ref.)
Single^5^	788	16.6	4016	16.9	0.98	0.89–1.07
Divorced	827	17.4	4152	17.4	0.99	0.91–1.08
Widowed	146	3.1	669	2.8	1.09	0.91–1.31
*No of people in the household*						
1	1050	22.1	5068	21.3	1.00	(ref.)
2 or more	3711	78.0	18737	78.7	0.96	0.89–1.03
Total no.	4761		23805			

1 Working or studying at least part time. Self-employed included.

2 Includes all women with disability pension at least part-time and/or with sickness benefit.

3 Including women who part time registered as unemployed or more.

4 Social welfare allowance (own) and/or housing allowance (own).

5 Includes cohabitants without registered partnership.

#### Outcome

Due to the social insurance system in Sweden (see above) it is difficult to use either employment or income as an outcome variable. A person could be registered as working full-time if he/she is on sick-leave up to 100% from full-time work. Also, the disposable income (up to a certain income level as mentioned above) is not affected to a great extent. We therefore chose to use sickness absence as our main outcome variable.

### Statistical methods

In order to compare women diagnosed with breast cancer with their matched controls 1 year prior to diagnosis, percentages in various categories of the following variables were compared: education, working at least part time, sickness absence, unemployment, retirement, welfare, disposable income, marital status, and number of people living in the household. To quantify the differences between the two groups conditional logistic regression was used to account for the matching with differences reported as odds ratios (OR) with 95% confidence intervals (CI). Only unadjusted estimates are reported, as the main interest was to investigate whether there were any imbalances between the groups before the women with breast cancer had been diagnosed (Table 2).

In order to investigate differences in working life, income and marital status in women diagnosed with breast cancer with their matched controls at 3 and 5 years post diagnosis, conditional Poisson regression was used to estimate a risk ratio (RR) with a 95% CI. A separate model was fitted for each factor of interest with each model adjusted for education level and the factor of interest recorded 1 year prior to diagnosis (Table 3).

**Table 3:** Working life, income and marital status 3 and 5 years following a breast cancer diagnosis between 1993 and 2003 and matched women without breast cancer.

		3 years after diagnosis				5 years after diagnosis			
				Adjusted model^6^				Adjusted model^6^	
		No./Total	%	RR	95% CI	No./Total	%	RR	95% CI
Working (part-time or more)^1^	Not breast cancer	19555/23805	82.2	1.00	(ref.)	14761/18875	78.2	1.00	(ref.)
	Breast cancer	3860/4761	81.1	0.97	0.94–1.01	2881/3775	76.3	0.96	0.93–1.00
Sickness benefit	Not breast cancer	4698/23805	19.7	1.00	(ref.)	3708/18875	19.7	1.00	(ref.)
	Breast cancer	1398/4761	29.4	1.49	1.40–1.58	917/3775	24.3	1.24	1.15–1.33
Disability pension	Not breast cancer	4597/23805	19.3	1.00	(ref.)	4100/18875	21.7	1.00	(ref.)
	Breast cancer	1168/4761	24.5	1.47	1.37–1.58	1103/3775	29.2	1.47	1.37–1.58
Sickness absence^2^	Not breast cancer	8384/23805	35.2	1.00	(ref.)	7035/18875	37.3	1.00	(ref.)
	Breast cancer	2204/4761	46.3	1.36	1.30–1.43	1788/3775	47.4	1.31	1.24–1.38
Unemployed^3^	Not breast cancer	2893/23805	12.2	1.00	(ref.)	2093/18875	11.1	1.00	(ref.)
	Breast cancer	555/4761	11.7	0.95	0.86–1.05	376/3775	10.0	0.90	0.80–1.01
Welfare^4^	Not breast cancer	1584/23805	6.7	1.00	(ref.)	1004/18875	5.3	1.00	(ref.)
	Breast cancer	275/4761	5.8	0.98	0.85–1.13	165/3775	4.4	0.88	0.73–1.06
Income increase with	Not breast cancer	12517/23805	52.6	1.00	(ref.)	11941/18875	63.3	1.00	(ref.)
10% or more	Breast cancer	2414/4761	50.7	0.99	0.96–1.01	2344/3775	62.1	0.99	0.96–1.01
Income increase with	Not breast cancer	7714/23805	32.4	1.00	(ref.)	8944/18875	47.4	1.00	(ref.)
20% or more	Breast cancer	1459/4761	30.6	0.99	0.97–1.02	1701/3775	45.1	0.98	0.95–1.01
Are married^5^	Not breast cancer	14476/23805	60.8	1.00	(ref.)	11494/18875	60.9	1.00	(ref.)
	Breast cancer	2955/4761	62.1	1.02	0.98–1.07	2356/3775	62.4	1.02	0.97–1.07
Divorced	Not breast cancer	4630/23805	19.5	1.00	(ref.)	3721/18875	19.7	1.00	(ref.)
	Breast cancer	889/4761	18.7	0.95	0.87–1.05	720/3775	19.1	1.00	0.90–1.10
Single household	Not breast cancer	6111/23805	25.7	1.00	(ref.)	5144/18875	27.3	1.00	(ref.)
	Breast cancer	1209/4761	25.4	0.95	0.88–1.02	1013/3775	26.8	0.94	0.88–1.02

1. Working or studying at least part time. Self-employed included.

2. Includes all women with disability pension at least part-time and/or with sickness benefit.

3. Including women who are part time registered as unemployed or more.

4. Social welfare allowance (own) and/or housing allowance (own).

In order to compare the effect of stage at diagnosis and treatment on sickness absence at 3 and 5 years post diagnosis for women diagnosed with breast cancer, unconditional logistic regression was used with differences reported as odds ratios with 95% CIs. The following factors were investigated, working 1 year before diagnosis, education, tumour size, having lymph nodes, sentinel node, stage, and treatment. For each variable two models were compared; the first model adjusted for education and the factor of interest one year prior to diagnosis and the second model adjusted for education and the factor of interest one year prior to diagnosis and the remaining investigated factors (Table 4).

**Table 4:** Odds ratios (OR) and 95% confidence intervals (CI) of sickness absence 3 and 5 years following a diagnosis of stage I-IIb breast cancer.

	Sickness absence after 3 years						Sickness absence after 5 years					
	Yes	Model 1		Model 2		Yes	Model 3		Model 4	
	No./Total	%	OR	95% CI	OR	95% CI	No./Total	%	OR	95% CI	OR	95% CI
*Sickness absence 1 year prior*												
Yes	933/1169	79.8	1.00	(ref.)	1.00	(ref.)	685/868	78.9	1.00	(ref.)	1.00	(ref.)
No	946/2925	32.3	0.12	0.10–0.15	0.12	0.10–0.14	863/2375	36.3	0.16	0.13–0.19	0.15	0.13–0.19
*Education 1 year before*												
Low	528/1006	52.5	1.00	(ref.)	1.00	(ref.)	461/841	54.8	1.00	(ref.)	1.00	(ref.)
Middle	857/1881	45.6	0.88	0.75–1.05	0.89	0.75–1.06	691/1451	47.6	0.85	0.71–1.03	0.86	0.71–1.03
High	490/1197	40.9	0.84	0.70–1.02	0.83	0.68–1.00	394/943	41.8	0.77	0.63–0.94	0.76	0.61–0.93
Unknown	4/11	36.4	0.57	0.14–2.25	0.49	0.12–2.00	2/8	25.0	0.50	0.10–2.49	0.44	0.09–2.23
*Tumour size*												
1–10	336/819	41.0	1.00	(ref.)	1.00	(ref.)	284/639	44.4	1.00	(ref.)	1.00	(ref.)
11–20	907/2047	44.3	1.15	0.96–1.38	1.01	0.84–1.22	755/1632	46.3	1.09	0.90–1.34	1.02	0.83–1.26
21–50	610/1183	51.6	1.66	1.36–2.03	1.14	0.91–1.42	490/933	52.2	1.49	1.20–1.86	1.18	0.93–1.51
50+	26/46	56.5	2.19	1.15–4.18	1.47	0.76–2.88	19/39	48.7	1.25	0.62–2.50	0.98	0.48–2.02
*Lymph nodes*												
N0	1189/2732	43.5	1.00	(ref.)	1.00	(ref.)	992/2165	45.8	1.00	(ref.)	1.00	(ref.)
N+ (Yes)	690/1363	50.6	1.49	1.29–1.72	1.00	0.83–1.21	556/1078	51.6	1.36	1.16–1.59	1.08	0.88–1.33
*Sentinel node*												
Sentinel node only	116/278	41.2	1.00	(ref.)			25/56	44.6	1.00	(ref.)		
Axillary dissection (1–9)	661/1525	43.3	1.26	0.94–1.69			630/1313	48.0	1.41	0.78–2.54		
Axillary dissection (10+)	1096/2279	48.1	1.59	1.20–2.12			889/1863	47.7	1.44	0.80–2.59		
Missing	6/13	46.2	1.18	0.33–4.24			4/11	36.4	0.68	0.15–3.04		
*Surgery*												
Mastectomy	629/1193	52.7	1.00	(ref.)	1.00	(ref.)	503/955	52.7	1.00	(ref.)	1.00	(ref.)
Breast-conserving	1241/2881	43.7	0.62	0.53–0.72	0.71	0.59–0.86	1038/2272	45.7	0.72	0.61–0.85	0.77	0.63–0.95
None or missing	9/21	42.9	-	-	-	-	7/16	43.8	-	-	-	-
*Received radiotherapy*												
Yes	1605/3539	45.4	0.90	0.74–1.10	0.99	0.78–1.26	1332/2796	47.6	1.03	0.83–1.28	1.12	0.86–1.44
No	274/556	49.3	1.00	(ref.)	1.00	(ref.)	216/447	48.3	1.00	(ref.)	1.00	(ref.)
*Received chemotherapy*												
Yes	777/1483	52.4	1.74	1.51–2.01	1.55	1.29–1.86	558/1083	51.5	1.42	1.22–1.67	1.23	1.00–1.50
No	1102/2612	42.2	1.00	(ref.)	1.00	(ref.)	990/2160	45.8	1.00	(ref.)	1.00	(ref.)
*Received hormonal therapy (Tamoxifen)*												
Yes	822/1629	50.5	1.35	1.17–1.55	1.25	1.08–1.46	537/1055	50.9	1.20	1.02–1.40	1.08	0.90–1.29
No	1057/2466	42.9	1.00	(ref.)	1.00	(ref.)	1911/2188	46.2	1.00	(ref.)	1.00	(ref.)

Model 1: Adjustment for sickness absence 1 year before diagnosis, education.

Model 2: Adjustment for sickness absence 1 year before diagnosis, education, tumour size, having lymph nodes, stage, and treatment.

Model 3: Adjustment for sickness absence 1 year before diagnosis, education.

Model 4: Adjustment for sickness absence 1 year before diagnosis, education, tumour size, having lymph nodes, stage, and treatment.

## Results

### Differences 1 year prior to diagnosis (Table 2)

Compared to women with low education, the risk of breast cancer was higher for women with high (OR = 1.21, 95% CI 1.11–1.32) and middle (OR = 1.13, 95% CI 1.05–1.23) education. Also, the risk of a breast cancer diagnosis was somewhat higher among women who worked (OR = 1.14, 95% CI 1.04–1.26), and lower among women who had received welfare allowance (OR = 0.87, 95% CI 0.78–0.97) (Table 2). No significant associations were found for sickness benefit, disability pension, unemployment, income, marital status or household size (Table 2).

### Differences 3 and 5 years after the diagnosis (Table 3)

Breast cancer had a post diagnostic effect both 3 and 5 years after the diagnosis on sickness benefits and disability pension (Table 3). In the third year after diagnosis, a larger proportion of women with breast cancer had received sickness benefit compared to women without breast cancer (risk difference = 9.7%, RR = 1.49, 95% CI 1.40–1.58), or had received disability pension (risk difference = 5.2%, RR = 1.47, 95% CI 1.37–1.58 (Table 3). The same pattern was present after 5 years, with a larger proportion of women with breast cancer, compared to women without breast cancer, receiving sickness benefit (risk difference = 4.6%, RR = 1.24, 95% CI 1.15–1.33), or disability pension (risk difference = 7.5%, RR = 1.47, 95% CI 1.37–1.58), although the difference in sickness benefit had decreased and the difference in disability pension had increased (Table 3). We found no influence of breast cancer on income, welfare use or marital status either 3 or 5 years after diagnosis, whereas breast cancer had a borderline effect on working part-time or less (Table 3).

### Sickness benefit and disability pension in relation to stage at diagnosis (Figure 1)

**Figure 1:**
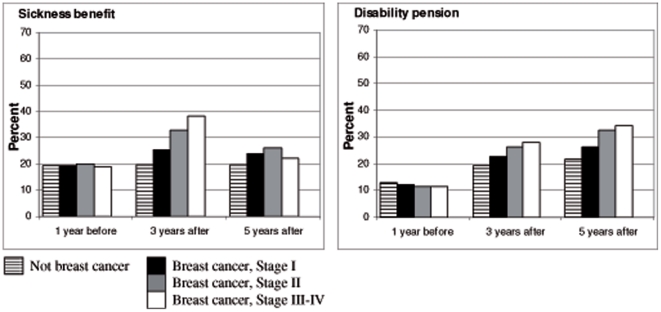
Proportion of women, with and without breast cancer, who received sickness benefit or disability pension 3 and 5 years following a diagnosis.

Figure 1 displays the effect of a breast cancer diagnosis stratified by tumour stage on sickness benefit and disability pension 3 and 5 years after diagnosis. There was a statistically significant effect of breast cancer on sickness benefit and disability pension in all stages, but the effect seemed to be stronger in more advanced stages. Also the effect of sickness benefit was much greater at 3 years compared to 5 years after diagnosis, whereas the effect on disability pension had increased after 5 years.

The proportion of women who had received sickness benefit was after 3 years 19.2% among women without breast cancer, compared to 25.3%, 33.0%, and 38.2% in women with breast cancer stage I, II, III-IV, respectively and after 5 years 19.7% among women without breast cancer, compared to 23.9%, 26.2%, and 22.1% in women with breast cancer stage I, II, III-IV, respectively.

The proportion of women with disability pension was after 3 years 19.3% among women without breast cancer, compared to 22.6%, 26.1%, and 28.0% in women with breast cancer stage I, II, III-IV respectively, and after 5 years 21.7% among women without breast cancer, compared to 26.1%, 32.5%, and 34.3% in women with breast cancer stage I, II, III-IV, respectively (Figure 1).

### Sickness absence among women with early stage breast cancer (stage I-IIb) in relation to tumour characteristics and treatment (Table 4)

#### 3 years after diagnosis

After adjustment for sickness absence and education 1 year prior to the diagnosis, the likelihood for women with stage I-IIb disease to be on sick leave was positively associated with larger tumours, lymph node metastasis, treatment by mastectomy and receiving adjuvant chemo- or hormonal therapy, whereas treatment by radiotherapy had no effect (Model 1, Table 4). After adjustment for treatment neither tumour size nor lymph nodes had any effect (Model 2, Table 4).

#### 5 years after diagnosis

The effects of tumour characteristics and treatment were somewhat weaker compared to those after 3 years except that of education. The likelihood of women with stage I-IIb disease to be on sickness absence was positively associated with a low educational level, large tumours, lymph node metastasis and treatment by mastectomy, chemotherapy or hormonal therapy, whereas treatment by radiotherapy had no effect (Model 3, Table 4). After adjustment for treatment neither tumour size, the presence of lymph nodes metastasis or hormonal therapy had any significant effect (Model 4, Table 4).

#### Chemotherapy and Hormonal treatment in relation to type of sickness absence

After adjustment for tumour characteristics, treatment by chemotherapy increased the risk for sickness benefit in the 3'rd but not in the 5'th year after diagnosis (RR = 1.67, 95% CI 1.40–2.00, and RR = 1.10, 95% CI 0.89–1.36 respectively), but had no effect on the risk for disability pension either 3 or 5 years after diagnosis (RR = 1.10, 95% CI 0.86–1.41, and RR = 1.24, 95% CI 0.98–1.57 respectively). Hormonal treatment also increased the risk for sickness benefit 3 but not 5 years after diagnosis (RR = 1.21, 95% CI 1.04–1.41, and RR = 0.93, 95% CI 0.76–1.12 respectively), but did, in contrast to chemotherapy, have an effect on the risk for disability pension both 3 or 5 years after diagnosis (RR = 1.47, 95% CI 1.19–1.80, and RR = 1.40, 95% CI 1.13–1.72 respectively) (data not shown).

*Sentinel node:* The risk of sickness absence was higher among patients with stage I-IIb breast cancer who had had axillary lymph node dissection compared to sentinel node biopsy (Table 4).

## Discussion

Although many cancer survivors are able to return to a normal life and functioning following treatment, many women of working ages do not. We found that a breast cancer diagnosis was associated with an increased risk of at least part time sickness benefit or disability pension even after 5 years, although the effect on sickness benefit was weaker after 5 years. This pattern was evident for all stages of disease at time of diagnosis, although it was somewhat less pronounced in early stage disease. The negative influence on working life was greater among women who had undergone surgery by mastectomy or received chemotherapy or hormonal treatment, treatment modalities which have known side-effects. All observed associations were weaker after 5 years compared to 3 years.

Strengths of the present study included the design with cases identified in a large population based clinical register and the use of matched controls in order to minimize confounding of the socioeconomic variables, and also the baseline determination of outcome variables at the age of one year before breast cancer diagnosis for the cases. Weaknesses included absence of information on recurrences and that the information in the clinical register is limited to intended chemo- and hormonal treatment, with no data available on actual treatment received. Especially since it is known that adherence to hormonal therapy often is lower than expected (65–85%) [27].

Contrary to findings in earlier studies[9], we did not find any effect on working after 3 or 5 years between survivors of breast cancer and controls. However, this could be explained by that the working variable used cannot reveal whether an individual returns to work to the same degree as she had before diagnosis, but only that she works at least part of the time. When we used sickness benefit and disability pension as endpoints, we found large differences between cancer survivors and women without breast cancer. Another Nordic study also indicated an increased risk of early retirement among breast cancer survivors[12]. One obvious reason for the detected differences on our study could be the effects of advanced disease on future life situation, but we also found differences among women diagnosed with early stage breast, a group with a higher disease-free survival.

In contrast to other investigations[13], [14], [28] we did not find that unemployment, use of welfare, or lower income was more common among breast cancer survivors. However, a possible explanation, at least in part, could be the Swedish social insurance system, which guarantees a right to receive some sort of sick compensation and that illness in itself does not constitute grounds for dismissal according to Swedish legislation. Also, the disposable income includes incomes from different sources (both earned by employment, unemployment benefit, sickness benefit and insurances and so forth) after taxation, making it impossible to investigate any effects on labour earnings only. As reported by others[19], [20], change of marital status or divorce was not more common among breast cancer survivors.

The major determinants for sickness absence after 5 years were level of education and type of treatment. Low education, low socioeconomic standing and having a manual job have previously been reported to be risk factors for early retirement and unemployment after a cancer diagnosis[12], [13], although the risk of unemployment was estimated as small. Perhaps differences in the nature of working tasks, with more manual labour in lower socioeconomic groups, is one important explanation for the difference found between educational groups. Workplace accommodations have also been found to play an important role for returning to work after cancer treatment[10].

Treatment by mastectomy and axillary dissection were also risk factors for sickness absence after 3 and 5 years. Mastectomy has also been reported as being associated with an increased risk for chronic pain[29] or lymphoedema[30], compared to after breast conserving surgery. Studies have also found that physical and social function, general health[31], or role functioning[32] after a mastectomy remains lower 5 years after the surgery compared to after breast conserving surgery. Axillary lymph node dissection has known side-effects such as lymphoedema, restricted shoulder mobility, pain, and sensory disturbances. The risks of these side-effects have been shown to be smaller when using sentinel node biopsy[33], [34], [35], [36]. In contrast to others[37], we did not find any negative effect of receiving radiotherapy on sickness absence from work despite the well-known side-effects associated with radiotherapy towards the axilla and supraclavicular fossa[36].

Adjuvant chemotherapy, did however, negatively affect both sickness benefit and disability pension after 3 years, but not after 5 years. This finding corroborates results from other studies reporting short time effects of chemotherapy[15], [17], or that chemotherapy did not have any long time effect on quality of life[3]. A recent Danish study did not find any associations between chemotherapy and long term sequelae[37]. This implies that most of the side-effects of chemotherapy that could affect work (such as fatigue, nausea, and anxiety of this treatment) are transient.

Adjuvant hormonal therapy did have a persistent impact on disability pension even after 5 years, which is in line with prior investigations associated with returning to work [11], whereas the effect on sickness benefit decreased after 5 years. The Danish study found a correlation between endocrine therapy and symptoms affecting daily activities including stopping or changing work[37]. In Sweden, endocrine therapies are usually given for 5 years following the end of primary treatment. In other words, most women that were prescribed Tamoxifen in our study were still on this treatment during follow-up, making it difficult to examine possible post-treatment side-effects on working activity. However, extended periods for endocrine treatments underline the importance of investigating side-effects. Especially considering that the longer a person is away from work due to an illness, the harder it is to return to the labour market[38], [39]. Furthermore, even longer treatment than 5 years has been recommended[40] which mean that this question is of growing importance. The management of side-effects is not only important with regard to quality of life and working activity, but also in that it may influence adherence to treatment in a way that ultimately can compromise the chance of being cured of breast cancer.

### Interpretation

Even in early stage breast cancer, the diagnosis was negatively associated with sickness absence both 3 and 5 years after diagnosis, and was most pronounced in women who underwent mastectomy or received chemotherapy or hormonal treatment. The knowledge about how to increase the number of women returning to full-time work is rather unexplored, and a study has also found that many cancer patients report unmet rehabilitation needs[41]. A greater focus needs to be put on rehabilitation of breast cancer patients (such as oedema, pain, psychological distress), investigation of eventual positive effects of health-related lifestyle changes (such as physical activity and diet), physical work-place adaptations and research on long-term sequelae of treatment to receive a better understanding of the women's life-situation and to be able to target efforts.
